# Teratogenic Effect of Crude Ethanolic Root Bark and Leaf Extracts of *Rauwolfia vomitoria* (Apocynaceae) on Nissl Substances of Albino Wistar Rat Fetuses

**DOI:** 10.1155/2013/906731

**Published:** 2013-03-24

**Authors:** Mokutima A. Eluwa, Theresa B. Ekanem, Paul B. Udoh, Moses B. Ekong, Olaitan R. Asuquo, Amabe O. Akpantah, Agnes O. Nwakanma

**Affiliations:** ^1^Department of Anatomy, Faculty of Basic Medical Sciences, University of Calabar, PMB 1115 Calabar, Nigeria; ^2^Department of Zoology and Environmental Biology, Faculty of Natural and Applied Sciences, University of Calabar, PMB 1115 Calabar, Nigeria; ^3^Department of Anatomy, Faculty of Basic Medical Sciences, University of Uyo, PMB 1017 Uyo, Nigeria; ^4^Department of Anatomy, Faculty of Basic Medical Sciences, Anambra State University, Uli, Nigeria

## Abstract

*Rauwolfia vomitoria *is a plant used for the treatment of insanity. The possible adverse effects of crude ethanolic root bark and leaf extract of the plant on Nissl substances of albino Wistar rat fetuses were studied using 25 mature female Wistar rats. The animals were divided equally into 5 groups, labeled A, B, C, D, and E. Group A was the control, while groups B, C, D, and E were the experimental. The female rats were mated with the males overnight, and the sperm positive day was designated as day zero of pregnancy. Oral doses of 150 mg/kg and 250 mg/kg body weight of the root bark extract were administered to groups B and C animals, respectively, while groups D and E animals received 150 mg/kg and 250 mg/kg body weight of the leaf extract, respectively, from day 7 to 11 of gestation. On day 20 of gestation, the rats were sacrificed, the fetuses brains extracted, and the cerebral cortices excised and routinely processed for Nissl substances using Cresyl fast violet staining method. Results showed reduced staining intensity of Nissl substances in the treated groups, especially those that received the root extract. Thus, the herbs may have adverse effects on protein synthesis within the cerebral cortex.

## 1. Introduction

Herbal medicine is the oldest form of health care known to humanity and has been used in all cultures throughout history. Primitive people learned by trial and error to distinguish useful plants with beneficial effects from those that were toxic or nonactive. Even in cultures, tribal people methodically collected information on herbs and developed well-defined herbal pharmacopeias. Traditional medicine evolved over centuries depending on local flora, culture, and religion [1–3].

*Rauwolfia vomitoria,* the herb of study is mostly found in the rain forest of Southern Nigeria. The plant has many common names like serpent wood, serpent snake root, and swizzle stick. In Nigeria, it is known as asofeyeje in Yoruba, ira in Hausa, akata in Bini, and mmoneba and utoenyin in Efik languages, respectively [4]. Extensive studies carried out on its chemical properties showed that the plant contained more than 50 active indole alkaloids, each possessing remarkable pharmacological activities [5, 6].

Nigeria is one of the countries where traditional medicine practitioners prescribe and administer decoctions of the leaves and root bark to patients without regard to its possible adverse effects. This leads to the present investigation which was to assess the neurohistological effects of the crude ethanolic root bark and leaf extracts of *Rauwolfia vomitoria* (Apocynaceae) on Nissl substances in the cerebral cortex of albino Wistar rat fetuses.

## 2. Materials and Methods

Twenty-five adult female Wistar rats were bred in the animal house of the Department of Human Anatomy, University of Calabar Nigeria. They were fed with normal rat chow, and water was provided *ad libitum*  throughout the duration of the experiment. The rats were kept under standard room temperature of 25–27°C. The animals were divided into five groups designated as A, B, C, D, and E, each consisting of five rats. The group A animals were the control, and groups B, C, D, and E were the experimental animals.

### 2.1. Preparation of the Herb Extract

The roots and leaves of* Rauwolfia vomitoria* tree were collected from Ekpene Obo, Esit Eket Local Government Area, Akwa Ibom State, Nigeria and were identified and authenticated by the botanist in-charge of the botanical garden of the University of Calabar, Nigeria. The roots and the leaves were washed with water to remove the impurities. The roots bark were defoliated, dried in Carbolite moisture extraction drying oven (Grant Instruments, Cambridge, England) at 40°C–50°C as well as the leaf for 3 hours. The dried root bark and leaf were blended into powdered form using a Binatone kitchen blender and kept in glass containers with plastic cover. The extraction method involved cold ethanolic extraction, where a known weight of the blended sample was soaked in ethanol for 24 hours and then the extract was filtered and evaporated to dryness at room temperature to obtain the crude extract.

### 2.2. Experimental Protocol

The twenty-five virgin female Wistar rats were caged with sexually matured male rats of the same strain overnight after ascertaining the estrous phase of the estrous cycle. The presence of tailed structures in the vaginal smear the following morning confirmed coitus, and the sperm positive day was designated as day zero of pregnancy. 

Oral doses of 150 mg/kg and 250 mg/kg per body weight of ethanolic root bark and 150 mg/kg and 250 mg/kg per body weight of leaf extracts of *Rauwolfia vomitoria* were administered to pregnant rats in groups B, C, D, and E, respectively, on the 7th through the 11th day of gestation with the aid of an orogastric tube. The administration is shown in Table 1. The control, group A animals received corresponding volumes of distilled water on the corresponding days of gestation. 

The pregnancy was terminated on the 20th day of gestation by chloroform inhalation method and the fetuses were collected by uterectomy. The fetuses were blotted dry, examined for gross malformations, and weighed using Libror EB-330H sensitive balance. The brain was extracted by opening up the skull and preserved using 10% formaldehyde. Following complete fixation of the whole brain, the cerebral cortex was excised. Routine histological processing was carried out, and the brain sections were stained with Cresyl fast violet method for Nissl substance [7].

Images obtained from the slides were analysed to determine its staining intensity using “ImageJ” software. One-way analysis of variance was applied using “Primer” software, followed by a post hoc Students Newman-Keul test. Data were significant at *P* < 0.05.

## 3. Results

**Figure 1:**
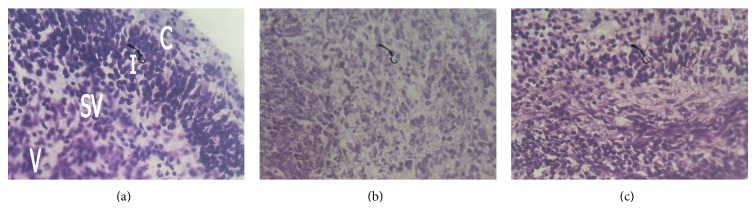
Photomicrographs of the cerebral cortex of control and treated group whose mothers received 150 mg/kg root bark and leaf extracts (Cresyl fast violet ×400 for all plates). (a) Control cerebral cortex section showing normal staining of Nissl substance with many neurons in the intermediate zone (I) and few in cortical (C), subventricular (SV), and ventricular layers (V). (b) Fetal cerebral cortex—150 mg/kg of root bark extract showing fewer neurons with reduced staining of Nissl substance. (c) Fetal cerebral cortex—150 mg/kg of leaf extract showing slight reduction in the staining intensity of the Nissl substance especially in the cortical layer.

**Figure 2:**
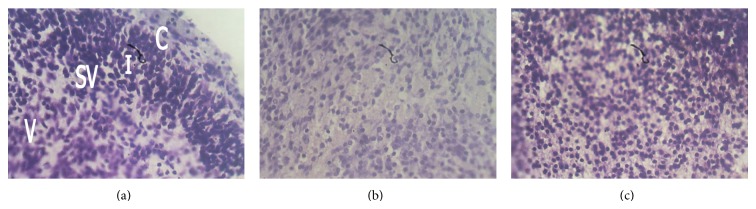
Photomicrographs of the cerebral cortex of control and treated group whose mothers received 250 mg/kg root bark and leaf extracts (Cresyl fast violet ×400 for all plates). (a) Control cerebral cortex section showing normal staining of Nissl substance with many neurons in the intermediate zone (I) and few in cortical (C), subventricular (SV), and ventricular layers (V). (b) Fetal cerebral cortex—250 mg/kg of root bark extract showing reduction in the staining intensity of Nissl substance. (c) Fetal cerebral cortex—250 mg/kg of leaf extract showing slight reduction in the staining intensity of the Nissl substance.

The Nissl substances of the control sections of the cerebral cortex were well stained (Figures 1 and 2(a)). Group B treated with 150 mg/kg of the root bark extract of *Rauwolfia vomitoria* showed reduced staining of Nissl substances (Figure 1(b)), while group D treated with 150 mg/kg of leaf extract of *Rauwolfia vomitoria* showed slight reduction in the staining intensity of Nissl substances (Figure 2(b)). Group C treated with 250 mg/kg of the root extract of *Rauwolfia vomitoria* showed reduction in staining intensity of Nissl substances (Figure 1(c)), while group E that received 250 mg/kg of the root extract of *Rauwolfia vomitoria* showed slight reduction in the staining intensity of Nissl substances when compared with the control (Figure 2(c)).

The staining intensity is shown in Table 2. The Nissl substance staining intensities in the cerebral cortex sections were not significantly (*P* = 0.904) different from the control.

## 4. Discussion

The neurons are the functional units of the nervous tissue and can be identified using special stains such as the Cresyl fast violet which hitherto was used to stain Nissl substances [7]. In this study, there were reductions in the staining intensities of Nissl substances in sections of the experimental groups when compared to the control. The reduction intensity was greater in groups B and C that received 150 mg/kg and 250 mg/kg, respectively, of the root bark extract when compared with groups D and E that received 150 mg/kg and 250 mg/kg of the leaf extract. The reduction in the staining intensities may be due to the adverse effects of the toxic constituent of *Rauwolfia vomitoria* root bark and leaf. It has been documented that chemicals and toxic substances affect the Nissl substance, thereby influencing their metabolic activity [8].

The decrease in Nissl substance staining may have been caused by chromatolysis. Chromatolysis is the migration of the Nissl substances towards the periphery of the soma due to either trauma or due to other exogenous agents [7, 9]. This usually results in the loss of function of the protein synthesizing ability of the neurons, and since protein is the working molecules of the cells, this may ultimately result in death of the cells. Our findings in this study are also in line with those of the work of Ajibade et al. [10], which showed that the Nissl substances in the cerebellar cortex in control rats stained more intensely compared with the less intense stain and degenerated Nissl substance in the treated rats following quinine administration.

Nissl substance is lost after cell injury. Neurons contain Nissl substance which is primarily composed of endoplasmic reticulum, with the amount, form, and distribution varying in different types of neurons [11]. 

This study suggests that the ethanolic root extract of *Rauwolfia vomitoria* is more teratogenic than the leaf extract and has potential to cause cerebral tissue damage, evidenced by the reduced staining intensity of Nissl substances.

## Figures and Tables

**Table 1:** Oral administrations of the control and the treatment groups.

Groups (5)	Treatments	Duration (days)
Control (A)	Distilled water	5
B	150 mg/kg per body weight of ethanolic root bark of *Rauwolfia vomitoria *	5
C	250 mg/kg per body weight of ethanolic root bark of *Rauwolfia vomitoria *	5
D	150 mg/kg per body weight of ethanolic leaf extracts of *Rauwolfia vomitoria *	5
E	250 mg/kg per body weight of ethanolic leaf extracts of *Rauwolfia vomitoria *	5

**Table 2:** Nissl substance staining intensities of the cerebral cortex sections in the control and the treatment group animals.

Groups (*n* = 5)	Treatments	Staining intensity (RGB)
Control (A)	Distilled water	136.34 ± 22.21
B	150 mg/kg per body weight of ethanolic root bark of *Rauwolfia vomitoria *	129.88 ± 12.94^ NS^
C	250 mg/kg per body weight of ethanolic root bark of *Rauwolfia vomitoria *	126.77 ± 19.34^ NS^
D	150 mg/kg per body weight of ethanolic leaf extracts of *Rauwolfia vomitoria *	150.744 ± 13.64^ NS^
E	250 mg/kg per body weight of ethanolic leaf extracts of *Rauwolfia vomitoria *	134.83 ± 21.39^ NS^

RGB: red + green + blue. Data presented as mean ± error of mean. ^NS^Not significantly different from the control group at *P* < 0.05.
